# Homonymous Superior Quadrantanopia due to Erdheim-Chester Disease with Asymptomatic Pituitary Involvement

**DOI:** 10.1155/2017/2807461

**Published:** 2017-05-23

**Authors:** Roaa Ridha Amer, Sara Mohammed Qubaiban, Eman Abdulkarim Bakhsh

**Affiliations:** ^1^King Saud bin Abdulaziz University for Health Sciences, Riyadh, Saudi Arabia; ^2^King Fahad Medical City, Riyadh, Saudi Arabia

## Abstract

Polyostotic sclerosing histiocytosis, also known as Erdheim-Chester disease (ECD), is a rare form of non-Langerhans histiocytosis. ECD has wide clinical spectrums which mainly affect skeletal, neurological, dermatological, retroperitoneal, cardiac, and pulmonary manifestations. Here we describe a case of ECD in a 45-year-old female who presented initially with bilateral knee pain and homonymous superior quadrantanopia progressed to ophthalmoplegia and complete visual loss of the left eye over a period of one year. Plain X-ray of both knees showed bilateral patchy sclerosis of the distal femur and upper parts of the tibiae. Initial brain magnetic resonance imaging (MRI) showed bilateral enhancing masses in the temporal lobes anterior to the temporal horns, thickening of the pituitary stalk, partially empty sella, and involvement of the left cavernous sinus one year later. Our case is a peculiar case of ECD initially presented with unilateral homonymous superior quadrantanopia due to involvement of the visual apparatus in the mesial temporal lobe which progressed to unilateral ophthalmoplegia and total visual loss secondary to involvement of the cavernous sinus. Thus, the diagnosis of ECD should be kept in mind in the presence of bilateral bone sclerotic lesions.

## 1. Introduction

Polyostotic sclerosing histiocytosis, also known as Erdheim-Chester disease (ECD), is a rare form of non-Langerhans histiocytosis. ECD was first described in 1930 as a rare type of Langerhans histiocytosis which is characterized by foamy macrophage accumulation, fibrosis, chronic inflammation, and multiorgan failure [1]. ECD has wide clinical spectrums which mainly affect skeletal, neurological, dermatological, retroperitoneal, cardiac, and pulmonary manifestations which could be life-threatening in some cases. Here we describe a case of ECD with central nervous system (CNS) involvement presenting as unilateral visual field deficit that progressed to total visual loss due to involvement of the temporal lobes and the cavernous sinus.

## 2. Case

A 45-year-old female referred to our tertiary care hospital complaining of bilateral knee pain associated with swelling, intermittent generalized body pain that affected her daily activity, progressive painless visual loss in the left eye, and mild headache for one year. She was treated in another hospital symptomatically for bone pain with no improvement. Her past medical history was irrelevant except for vitamin D deficiency.

**Figure 1:**
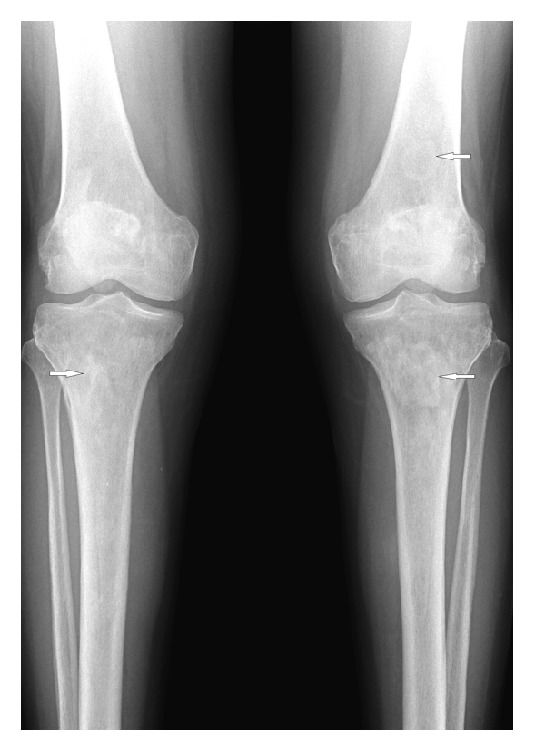
X-ray of both knees in anteroposterior view showing bilateral patchy high density donating sclerosis affecting the metaphysis of the distal femur and the proximal tibia bilaterally.

**Figure 2:**
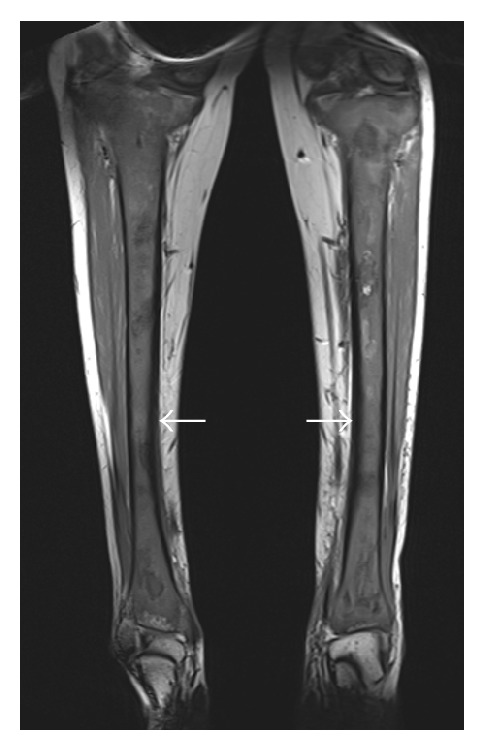
Bilateral tibial coronal T1 MRI showed bilateral asymmetrical hypointense lesions replacing the normal fatty signal intensity of both tibiae as compared to the normally appearing marrow fat signal in the talus. Note also the cortical thickening noted in the tibial cortices medially (arrows).

**Figure 3:**
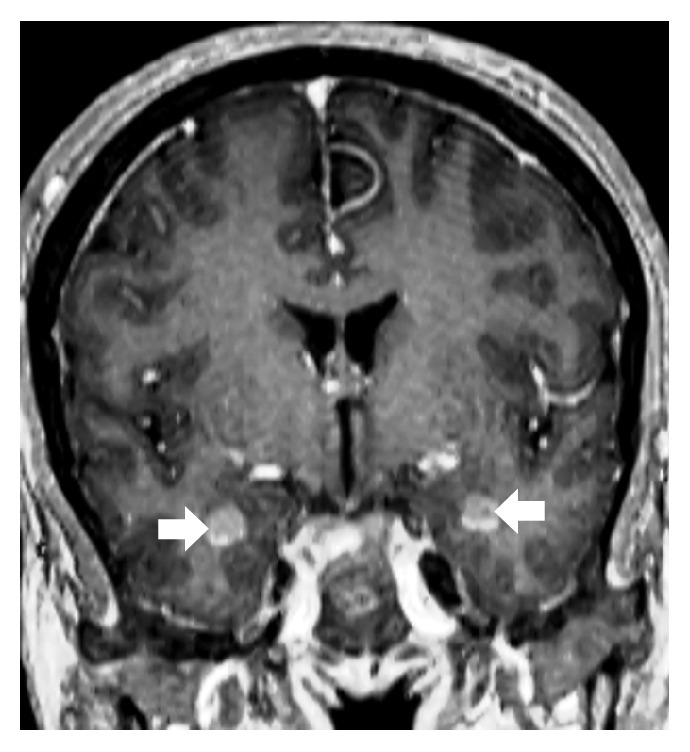
Contrast enhanced coronal T1 weighted image of the brain showing bilateral well-defined enhancing lesions in the temporal poles located laterally to the amygdala (bold arrows).

**Figure 4:**
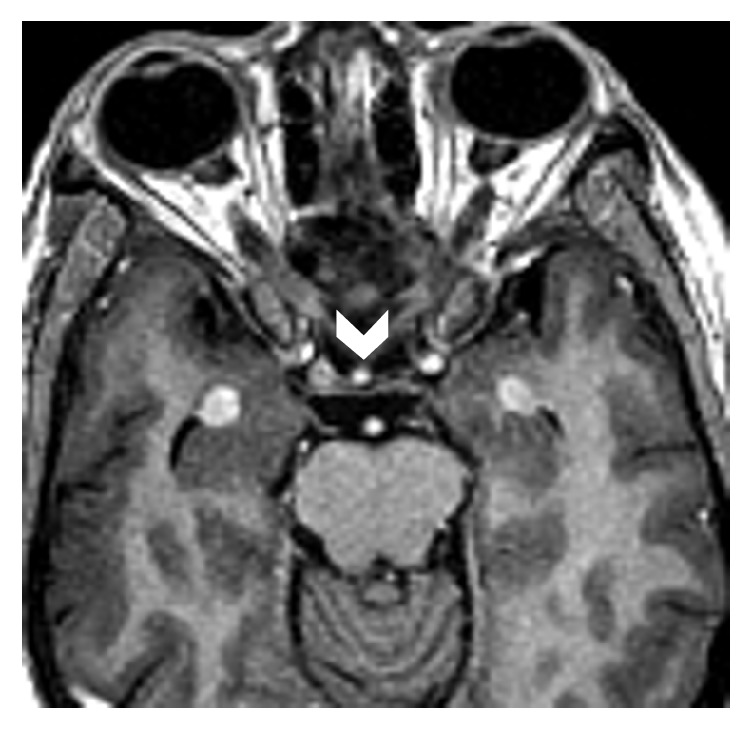
Postcontrast axial T1 weighted images of the brain at the level of the orbits showing the thickened enhanced pituitary stalk (arrow head) almost reaching the same size of the enhanced basilar artery. Note also the enhancing bilateral lesions anterior to the amygdala.

**Figure 5:**
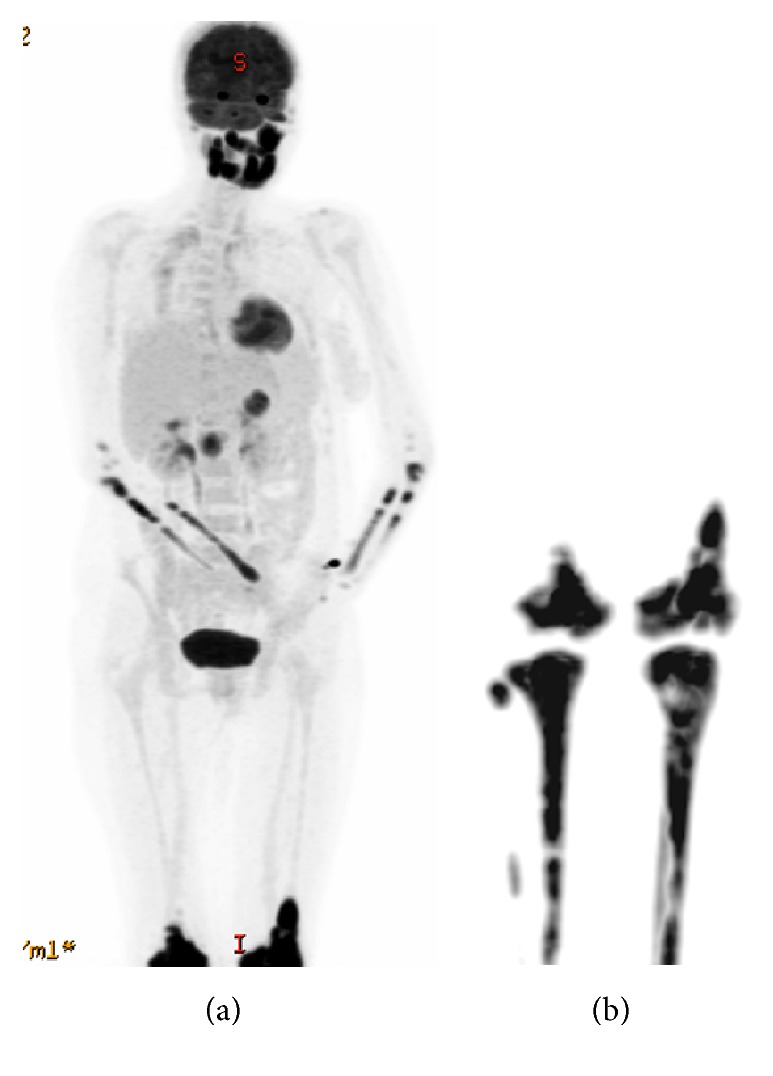
(a) FDG PET scan coronal reformate showing increased FDG uptake in temporal lobes, facial bones, radius, ulna, and distal femora. (b) FDG PET scan coronal reformatted images for the tibia showing bilateral increased FDG uptake in distal femora and tibiae.

On initial examination, she was afebrile, both knees were tender at joint line, with swelling over the anteromedial aspect of upper part of tibia with erythematous overlying skin. Patellar tap and ballottement tests as well as crepitus test were negative. Visual field testing revealed left homonymous superior quadrantanopia with normal visual acuity. Detailed slit lamp examination was insignificant and showed no involvement of retina. Complete blood count was normal at the time of presentation. Creatinine, urea, and electrolytes were within normal limits. High alkaline phosphatase and normal lactate dehydrogenase were present. Plain X-ray of both knees showed multiple sclerotic patches of the distal femur and upper parts of both tibiae (Figure 1) while bilateral tibia magnetic resonance imaging (MRI) showed bilateral lesions replacing the normal fatty signal intensity in the T1 weighted images (Figure 2). Initial brain MRI showed bilateral well-defined round intensely enhancing lesions located in the temporal poles lateral to the amygdala and anterior to the temporal horns with thickened enhanced pituitary stalk (Figure 3), in conjunction with partially empty sella and absent bright signal of the neurohypophysis (Figure 4). Whole body positron emission tomography (PET) scan showed increased FDG uptake in temporal lobes and facial and long bones (Figures 5(a) and 5(b)). She was further investigated for malignancies, paraneoplastic, autoimmune, neurodegenerative, and toxic causes. All tumor markers were within normal limits.

Left tibial biopsy was performed to rule out malignancy and showed marrow infiltration with sheets of foamy histiocytes with the presence of sclerosed trabeculae. The immunohistochemistry showed CD-1a negative histiocytes; however, molecular pathology showed negative BRAF mutation. The clinical, radiological, and immunohistochemical characteristics were consistent with ECD. The patient was started on standard interferon alpha 135 mg per week for a duration of 3 weeks and methylprednisolone with improvement of her symptoms after 1 week.

**Figure 6:**
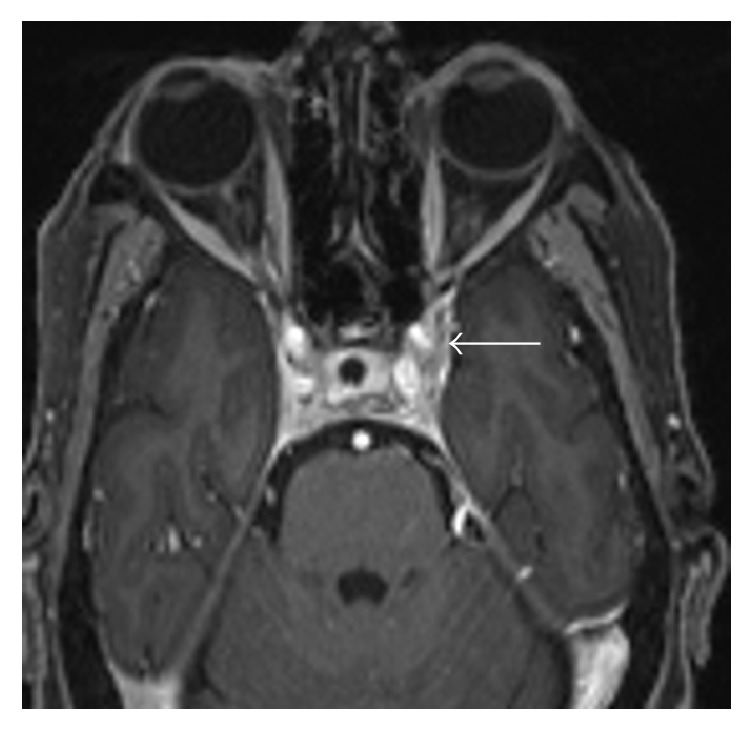
Axial contrast enhanced T1 weighted image of the brain showing increased outward convexity due to enhancing lesion in the left cavernous sinus (arrow) encasing the left internal carotid artery; however, it remained patent.

One year later, the patient presented with double vision and left ophthalmoplegia. On examination, she was found to have unequal pupils and papilledema. Brain MRI showed new enhancing lesion in the vicinity of the left cavernous sinus (Figure 6), with pachymeningeal thickening along the tentorial leaflets and interval regression of the mesial temporal lobe lesions.

## 3. Discussion

ECD is a rare myeloid neoplasm of multisystem involvement which can be potentially life-threatening [1–5]. Its etiology is unknown, and diagnosis is made based on the clinical, radiological, and histological characteristics. Only 500 cases of ECD were identified worldwide. There are no sufficient studies reporting the incidence of ECD in Saudi Arabia except for two case reports [6, 7]. Clinical presentation can vary from asymptomatic tissue infiltration and bone pain to multiorgan failure. ECD presents commonly with skeletal symptoms, diabetes insipidus (DI), ataxia, and constitutional symptoms [1].

Skeletal lesions are typically bilateral and symmetric, involving mainly lower limb bones. These lesions are usually found to be localized in the diaphysis-metaphysis junction and sparing the epiphysis and the axial skeleton [8–10]. Bone scans showed symmetric bilateral metadiaphyseal tracer uptake. This is usually manifested as chronic, constant, and localized bone pain associated with edema [1]. This is the most common presenting symptom in ECD accounting for 96% of cases [1]. These radiological findings are considered typical of ECD. Cranial bone involvement has been infrequently reported; however, it was described as osteosclerosis of maxillary and sphenoid sinuses which will demonstrate thickened bone on CT and hypointense signal on T1 and T2 weighted MRI images. Our patient had involvement of long bones as well as cranial bones.

CNS involvement is seen in about 40–50% of cases and responsible for 29% of all deaths due to the disease [11]. The most common locations are hypothalamopituitary axis, brain parenchyma, and meninges [11]. The neurological symptoms, in lowering order of frequency, are diabetes insipidus, exophthalmos, cerebellar ataxia, panhypopituitarism, and papilledema which correlate with the various radiological finding [11, 12]. Most common CNS radiological findings are involvement of the hypothalamic-pituitary axis where nodular or micronodular masses of the infundibular stalk may be present, retroorbital masses, involvement of the dentate area of the cerebellum, and meningeal lesions of the dura [13]. Other infrequent involvements include thickening of the bones of the face and skull, intracranial periarterial infiltration, intraluminal involvement of the superior sagittal sinus, involvement of the choroid plexus, and masses involving the cerebral hemispheres [13]. Most of the parenchymal lesions are found in the infratentorial region (brain stem, cerebellum, and middle cerebellar peduncles) which were not present in our case [13].

Approximately, 60% of patients will have simultaneous involvement of at least two CNS anatomical sites [13]. The involvement of the mesial temporal lobes in close proximity to the visual apparatus would explain the initial visual manifestation in our case. Although brain MRI revealed bilateral temporal lobes lesions, our patient was complaining of left homonymous superior quadrantanopia which could be explained by incomplete involvement of the visual apparatus on the unaffected side. Despite treatment our patient's visual symptoms relapsed with left total visual loss and ophthalmoplegia due to involvement of the left cavernous sinus. Furthermore, thickening of the pituitary stalk with loss of neurohypophysis bright spot and partially empty sella was noted on the initial MRI; however, no clinical manifestations of DI or hormonal disturbances were apparent as reported in literature.

## 4. Conclusion

Our case is a peculiar case of ECD involving the mesial temporal lobe which is a rare intracranial site as a cause of visual symptoms in ECD. Thus, ECD should be considered in the differential diagnosis of parenchymal intracranial lesions in the presence of bilateral skeletal sclerotic lesions.

## References

[1] Estrada-Veras J., O’Brien K., Boyd L. (2017). The clinical spectrum of Erdheim-Chester disease: an observational cohort study. *Blood Advances*.

[2] Veyssier-Belot C., Cacoub P., Caparros-Lefebvre D. (1996). Erdheim-Chester disease. Clinical and radiologic characteristics of 59 cases. *Medicine*.

[3] Gomez C., Diard F., Chateil J. F., Moinard M. (1996). Imagerie de la maladie d’Erdheim-Chester. *European Journal of Radiology*.

[4] Cattin F., Runge M., Nagy N. (2005). Case #5. Erdheim-Chester disease. *European Journal of Radiology*.

[5] Kenn W., Stäbler A., Zachoval R., Zietz C., Raum W., Wittenberg G. (1999). Erdheim-Chester disease: a case report and literature overview. *European Radiology*.

[6] Bohlega S., Alwatban J., Tulbah A., Bakheet S. M., Powe J. (1997). Cerebral manifestation of Erdheim-Chester disease: clinical and radiologic findings. *Neurology*.

[7] Binyousef R. F., Al-gahmi A. M., Khan Z. R., Rawah E. (2017). A rare case of Erdheim-Chester disease in the breast. *Annals of Saudi Medicine*.

[8] Breuil V., Brocq O., Pellegrino C., Grimaud A., Euller-Ziegler L. (2002). Erdheim-Chester disease: typical radiological bone features for a rare xanthogranulomatosis. *Annals of the Rheumatic Diseases*.

[9] Evans S., Williams F. (1986). Case report: Erdheim-Chester disease: polyostotic sclerosing histiocytosis. *Clinical Radiology*.

[10] Murray D., Marshall M., England E., Mander J., Chakera T. M. H. (2001). Erdheim-Chester disease. *Clinical Radiology*.

[11] Álvarez-Álvarez M., Macías-Casanova R., Fidalgo-Fernández M., Miramontes González J. (2016). Neurological involvement in Erdheim-Chester disease. *Journal of Clinical Neurology*.

[12] Sedrak P., Ketonen L., Hou P. (2011). Erdheim-Chester disease of the central nervous system: new manifestations of a rare disease. *American Journal of Neuroradiology*.

[13] Mazor R. D., Manevich-Mazor M., Shoenfeld Y. (2013). Erdheim-Chester disease: a comprehensive review of the literature. *Orphanet Journal of Rare Diseases*.

